# Organic compounds generated after the flow of water through micro-orifices: Were they synthesized?

**DOI:** 10.1016/j.heliyon.2017.e00376

**Published:** 2017-08-07

**Authors:** Tomiichi Hasegawa, Akiomi Ushida, Masaki Goda, Yasushi Ono

**Affiliations:** a Niigata College of Technology, 5-13-7 Kamishinei-cho, Nishi-ku, Niigata-shi, Niigata 950-2076, Japan; b Faculty of Engineering, Niigata University, 8050-2 Ikarashi, Nishi-ku, Niigata-shi, Niigata 950-2181, Japan; c Center for Research and Development in Natural Science, University of Toyama, 3190 Gofuku, Toyama-shi, Toyama 930-0887, Japan

**Keywords:** Physical chemistry, Organic chemistry, Analytical chemistry

## Abstract

Micro-fluid mechanics is an important area of research in modern fluid mechanics because of its many potential industrial and biological applications. However, the field is not fully understood yet. In previous work, when passing ultrapure water (UPW) in which air was dissolved (UPW*) through micro-orifices, we found that the flow velocity decreased and stopped over time, and membranes were frequently formed in the orifice when the flow stopped. The membrane came from the dissolved air in UPW*, and membrane formation was closely related to electric charges generated in orifices by the flow. In the present paper, we clarified the components of the membrane and suggested a mechanism for membrane formation. We examined the effect of contaminants on the membrane formation and confirmed our previous results. We identified the chemical components of the membrane and those present in the UPW* itself by using an electron probe microanalyzer and found that the proportion of each element differed between the membrane and UPW*. Raman and infrared (IR) spectroscopy showed that the membrane consisted of organic substances such as carotenoids, amides, esters, and sugars. We irradiated UPW* with ultraviolet light to cut organic chains that may be left in UPW* as contaminants. We found a similar membrane and organic compounds as in nonirradiated UPW*. Furthermore, although the UPW that was kept from contact with air after it was supplied from the UPW maker (UPW_0_) and bubbled with Ar gas (UPW_0_ bubbled with Ar) formed no membrane, the UPW_0_ bubbled with CO_2_ formed thin membranes, and Raman and IR analysis showed that this membrane contained carboxylic acid salts, carotenoids, or a mixture of both. We found that electric grounding of the orifice reduces the probability of membrane formation and that the jets issuing from an aperture bear negative charges, and we assumed that the micro-orifices possess positive charges generated by flows. Consequently, we suggest that organic compounds are synthesized from nonorganic matter in air or CO_2_ dissolved in water by the action of hydroxyl radicals generated by flows through micro-orifices.

## 1 Introduction

Micro-fluid mechanics is an important area of research in modern fluid mechanics because of its many potential applications in biology, medical science, engineering, and industry. However, flows through micro-orifices or short microtubes have been explored solely in relation to the flow properties [1], [2], [3], [4], [5], [6], [7], [8], [9], [10], [11], [12].

In a previous study [13], we measured the velocity (*V*) of ultrapure water (UPW, hereafter used as having the general meaning of ultrapure water) through a 20-μm orifice under a constant applied pressure ($P_{t}$) of between 50 and 1000 Pa for several types of UPW: UPW in which air was dissolved (UPW*), which was stored in a beaker after it was supplied by the UPW maker and exposed to air for more than 1 day; UPW* from which air was extracted by vacuum (deaerated UPW*); UPW that was kept from contact with air after it was obtained from the UPW maker (UPW_0_); and UPW_0_ that was bubbled with Ar gas (UPW_0_ bubbled with Ar). We found that *V* decreased for all types of water tested with elapsed time, and finally, the flow stopped. We removed the orifice in which the flow stopped from the experimental apparatus, observed it by using phase contrast microscope (PCM; Wraymer Inc., USA) and scanning electron microscopy (SEM; EPMA-1610, Shimadzu Corp., Japan), and found that a membrane frequently existed in the orifice. We found that the membrane comes from dissolved air. Membrane formation was found to decrease in an orifice that was electrically grounded, which means that electric charges are closely related to membrane formation. The detailed results have been published elsewhere [13].

In the current study, we clarified the components of the membrane and suggested a mechanism for membrane formation. We confirmed that dissolved air in water is the origin of the membrane by further examining leaching from the acrylic resin of the apparatus, organic matter originally present in the water, and dissolved air. We clarified that the charges affecting membrane formation were negative. We examined the components by using an electron probe microanalyzer (EPMA; EPMA-1610, Shimadzu Corp., Japan) and the compounds by observing Raman and infrared (IR) spectra for membranes formed in UPW*, UPW* irradiated with ultraviolet (UV) light (UV-irradiated UPW*), and UPW_0_ with dissolved CO_2_. We discussed where the membrane in the UPWs came from and suggested that organic compounds produced from air or CO_2_ in water via the action of hydroxyl radicals generated by flows through micro-orifices are responsible.

To our knowledge, the chemical components and origin of the membranes generated in micro-orifices after the flow stops have not been reported previously. However, we discuss several articles that are related to our conclusions.

## 2 Experimental

**Figure 1:**
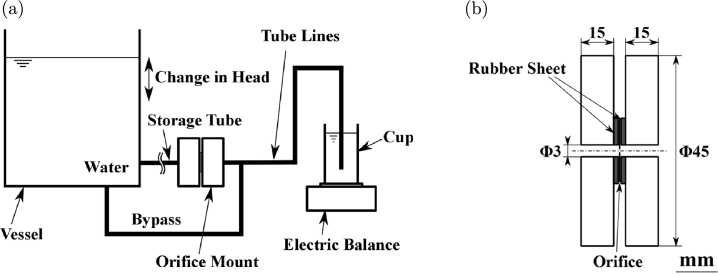
(a) Schematic of experimental setup for measuring flow rate and pressure in an orifice. (b) Orifice mount. The orifice was sandwiched between fluorocarbon rubber sheets, installed in an orifice mount, and connected to the vessel and the cup on the balance by a Tygon tube.

Water was allowed to flow through the orifices under pressures of 50, 100, 200, 500, and 1000 Pa applied by a head between a vessel and a cup during each experiment with the experimental setup (Figure 1(a)). The cross-sectional area was $200\;{\text{cm}}^{2}$ for the vessel and $30\;{\text{cm}}^{2}$ for the cup, which generated a head of about 0.1 mm (1 Pa) after the experiment. The pressure loss in the tube lines connecting the orifice, vessel, and cup was negligible compared with the pressure drop in the orifice. The orifice mount is shown in Figure 1(b). The flow rate was calculated by collecting water in the cup over time and weighing it on a balance (Shimadzu Corp.). The velocity was less than 1.0 m/s, and the maximum Reynolds number was 20. The orifices used were 20 μm in diameter (*D*) and 20 μm thick, and made of Ni or Ti. The experimental results did not differ greatly according to orifice material. The orifices have been described in detail in a previous paper [13]. Another experimental setup based on the same measuring method was used for providing a simple flow in front of the orifice [13]; however, there was no difference in the experimental results from the different setups.

UPW*, UPW_0_, and other treated UPWs were used, and tap water was used for comparison. All the test fluids were passed through 0.2-μm filters before the experiments. The temperature of the water was 18–25°C, and this variation in temperature had little effect on the flow.

Flow data were generally obtained over 4 days to examine the change in flow rates over the time, *t*, elapsed since the start of the flow experiment. One flow pass yielded five data points under the five pressures at a given *t*. More than six passes were usually conducted over 4 days in one experiment; thus, more than 30 data points were provided by one experiment. One pass took about 30 min and the flow channel was closed immediately after one pass until the next pass; no flow was provided to the orifice after the end of one pass until the start of the next pass.

We inspected the orifice after the flow stopped and examined the membranes that were frequently found in the orifice by PCM and SEM. Furthermore, we clarified the chemical components and structure of the membrane by PCM, SEM, and EPMA and by Raman and IR spectroscopy. We discussed where the components of the membrane came from and how the membrane was formed.

## 3 Results

### 3.1 Flow properties and membrane formation

**Figure 2:**
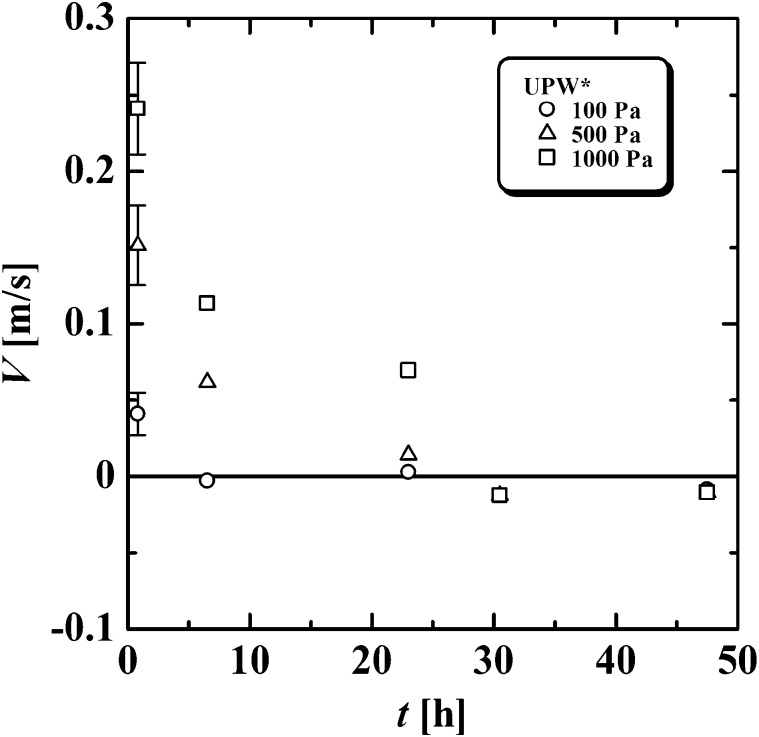
Flow properties of UPW*. Velocity, *V* (m/s), against elapsed time, *t* (h), for each pressure, *p* (Pa), for UPW* in the Ni orifice.

Figure 2 shows a typical example of the velocity, *V*, of UPW* through the Ni20 orifice as a function of *t* (h) for *p* at 100, 500, and 1000 (Pa). The range of experimental error is indicated by error bars in the figure. *V* gradually decreased with *t* and reached nearly zero at t=30 h. For example, at p=1000 Pa, V=0.24 m/s at t=0.8 h and V=0.11 m/s at t=6.5 h. Although there was no flow between t=0.8 and 6.5 h, *V* decreased from 0.24 to 0.11 m/s, suggesting that some factor reduced the flow for 5.7 h. Greater detail about the flow properties has been reported in a previous paper [13].

The orifices were removed from the apparatus after the flow stopped and examined from the SEM image acquired using the EPMA. Membranes were frequently found to form in the orifice hole [13]. Table 1 shows the probability of membrane formation, m/N, where *m* is the number of experiments in which the membrane formed in about 4 days, and *N* is the total number of experiments conducted for a given type of water. m/N of UPW*, 0.73, is larger than that of tap water, 0.28. This means that membranes were formed more frequently in the flow of UPW* than in that of tap water.

**Table 1:** Probability of membrane formation, *m*/*N*.

	UPW*	Tap Water
*m*	35	7
*N*	48	25
*m*/*N*	0.73	0.28

### 3.2 Chemical components of the membrane

#### 3.2.1 Mapping images with an EPMA

**Figure 3:**
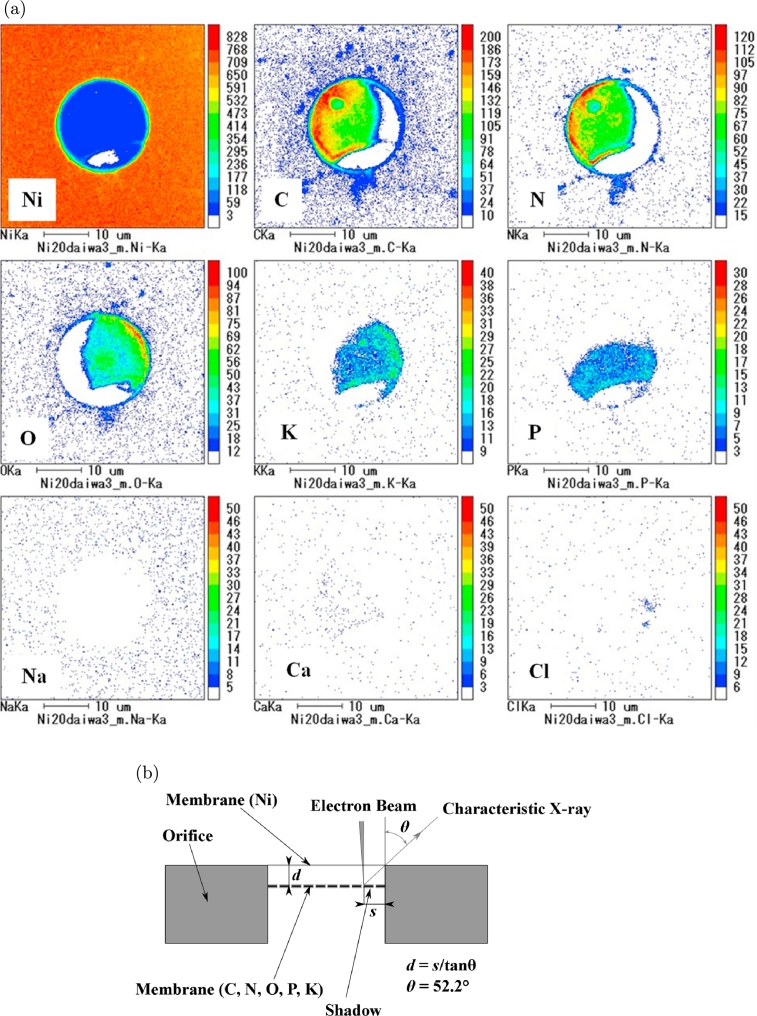
Mapping images. (a) Membrane from the Ni orifice and UPW*. This image corresponds to the SEM image in Figure 3(e)-1 in Ref. [13]. (b) Schematic of the two-layer membrane.

Figure 3(a) shows the mapping images of the orifice for UPW*, which corresponds to Figure 2. Ni was present over almost the entire orifice. This may be because Ni was dissolved in the UPW* and was incorporated into the membrane. C was seen over half the orifice. The white crescent on the right-hand side in the orifice was caused by the shadow of the characteristic X-rays, which were emitted at the upper right-hand side of the orifice at an angle of 52.5° from normal to the orifice plane. The white area near the bottom in the images in Figure 3(a) is attributed to membrane peeling [13]. A similar characteristic X-ray shadow was observed for *N*, although the shadow for O was on the opposite side because the emitted X-rays were detected in the opposite direction. There were low levels of K and P, although a shadow was observed on the left-hand side and at the top. The levels of Na, Ca, and Cl were below the detection limit. A schematic of the membrane was constructed from the mapping data (Figure 3(b)). The Ni layer, which showed no shadow, was in the same plane as the surface of the orifice, and another layer, which consisted of C, N, O, P, and K, was present below the surface. The distance, *d*, can be calculated from the length of the shadow from the orifice periphery, *s*, using the expression s/d=tan⁡θ. Figure 3(a) shows that s=5 μm and θ=52.5°; thus, *d* is 3.8 μm. The mapping images for the tap water membrane (not shown) were similar to those in Figure 3(a).

#### 3.2.2 Chemical components in membrane, UPW*, and tap water

We analyzed the elements in the membrane and compared them with those in UPW* and tap water. Quantitative analysis using an EPMA was conducted for the UPW* membrane (EPMA mapping is shown in Figure 3(a)), tap water membrane, UPW*, and tap water. The elements in the water samples were obtained by evaporating the water below 80°C to a dry residue. The air dissolved in the water samples was probably released because many bubbles were generated during evaporation. The mole percentages of the elements in each membrane and in the water samples are shown in Table 2. The concentrations of C and N were high in the membranes but low in the water samples; the UPW* membranes contained 60.7% C and 30.6% N, and the tap water membranes contained 52.9% C and 35.4% N. In contrast, the UPW* sample contained 2.7% C and 3.6% N, and the tap water sample contained 2.1% C and 5.9% N. The concentration of O was higher in UPW* and tap water compared with that in the membranes, which indicates that most elements existed as oxides in the water samples. Thus, the proportion of each element varied between the membranes and the water samples. This suggests that the membrane was not generated simply by the accumulation of the elements in water, but rather that it was generated by air dissolved in the water, although this was released during the evaporation of the sample.

**Table 2:** Chemical composition of UPW* membrane and UPW*, and tap water membrane and tap water.

	C	N	Ni	O	P	K	Si	S	Fe	Mg	Ca	Cl	Na
UPW* Membrane	60.7	30.6	2.5	3.8	0.9	0.3	0.4	0.3	0.1	0.3	0.1	0.1	–
UPW*	2.7	3.6	–	54.7	–	1.2	8.3	4.7	2.9	2.5	4.4	8.5	6.3

Tap Water Membrane	52.9	35.4	6.0	3.5	0.7	0.2	0.4	0.2	0.2	0.1	0.1	0.1	–
Tap Water	2.1	5.9	–	13.8	–	4.5	36.7	6.8	0.9	15.0	1.3	8.9	4.2

A further investigation revealed that the flow reduction and membrane formation did not occur when the water was flowing but did occur when the water was static after the flow was stopped. Therefore, the orifices did not accumulate substances that were originally present in the water via the flow. Rather, we propose that the membrane was produced by adsorption in still water after the flow stopped.

### 3.3 Origin of membrane components

There are three possible sources of the membrane material: material leached from the acrylic resin of the vessel, organic matter originally present in the water (Raman and IR spectroscopy show that the membrane contains organic matter), and air dissolved in water.

#### 3.3.1 Leaching from acrylic resin

**Figure 4:**
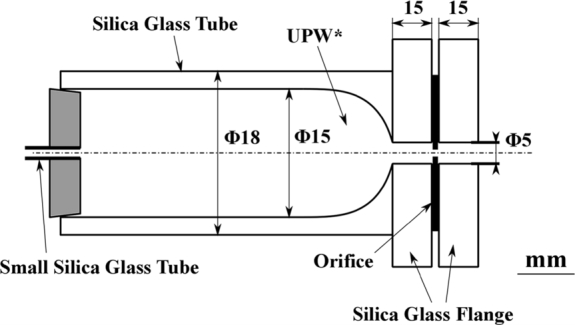
Silica glass tube. The orifice was attached to the end of the silica glass tube. The tube specifications are the same as those in Figure 8(b).

First, we examined whether material leached from the acrylic resin apparatus formed the membrane. The orifice was attached directly to the wall of the fluorocarbon resin vessel, which leaches very little into the water. However, the flow experiment produced a similar membrane. In addition, a similar membrane was generated when a silica glass tube, which does not leach organic material, was used as a reservoir for UPW* in front of the orifice (Figure 4). Therefore, even if material did leach from the acrylic resin, it had little effect on membrane formation.

#### 3.3.2 Organic matter originally present in the water

Next, organic matter originally present in the water was investigated. If the membrane formed from organic matter in the water, a membrane should be more easily produced in tap water than in UPW* because tap water contains more organic matter than UPW*. However, the probability of membrane formation, m/N, showed the opposite behavior (Table 1). The mass (*M*) of the UPW* membrane was estimated. The diameter of the membrane was 20 μm, its thickness was assumed to be 1 μm from the depth of the microscope focus, and its density was assumed to be $2\;\text{g}/{\text{cm}}^{3}$, which is typical for organic matter, and thus *M* was about $6.3\times 10^{-10}$ g. In contrast, the total organic carbon (TOC) in UPW was about $5\times 10^{-9}\;\text{g}/{\text{cm}}^{3}$. Therefore, the volume (*v*) of UPW* required to produce *M* was calculated as $0.13\;{\text{cm}}^{3}$. The volume ($v_{o}$) of the interior of the orifice was calculated as about $6\times 10^{-9}\;{\text{cm}}^{3}$, and $v/v_{o}=2.2\times 10^{7}$. This seems to be too large for membrane formation to be explained by adsorption of residual organic matter. However, if *M* is obtained from the dissolved air, *v* is calculated as $6.3\times 10^{-10}\;\text{g}/(2.3\times 10^{-5}\;\text{g}/{\text{cm}}^{3}\times 4\times 10^{-2})\approx 0.68\times 10^{-3}\;{\text{cm}}^{3}$, because the mass density of the dissolved air in water is $2.3\times 10^{-5}\;\text{g}/{\text{cm}}^{3}$ at 1 atm and 20°C and the ratio of carbon in air is about 0.04. The value of *v*, $0.68\times 10^{-3}\;{\text{cm}}^{3}$, is considerably lower than $0.13\;{\text{cm}}^{3}$ (the value for TOC in UPW), and $v/v_{o}=1.1\times 10^{5}$. This value is large but more realistic than $2.2\times 10^{7}$. The above considerations are not direct evidence supporting the notion that the organic matter left in water is not the source of the membrane. However, together with the data in Table 2, they suggest that the source is the air dissolved in water.

#### 3.3.3 Dissolved air

**Figure 5:**
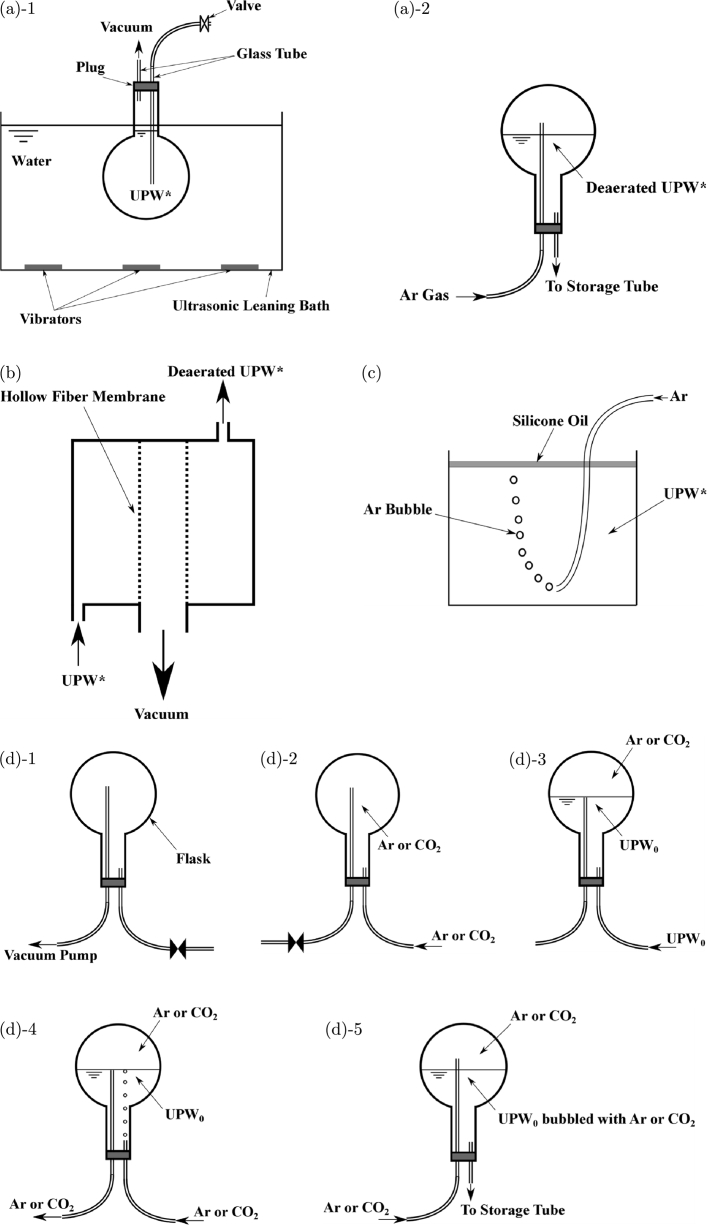
Deaeration and Ar bubbling of UPW*. (a)-1 Deaeration using vacuum and vibration. (a)-2 Deaerated UPW* obtained by method (a)-1 was sent to the storage tube by injecting Ar gas into the flask to prevent air contact. (b) Deaeration using a hollow fiber membrane and vacuum. (c) Ar bubbling. (d) More effective bubbling: (1) evacuating a flask using a vacuum pump, (2) filling flask with Ar or CO_2_, (3) injecting UPW_0_ into the flask, (4) bubbling with Ar or CO_2_, and (5) transporting the water to the storage tube.

**Figure 6:**
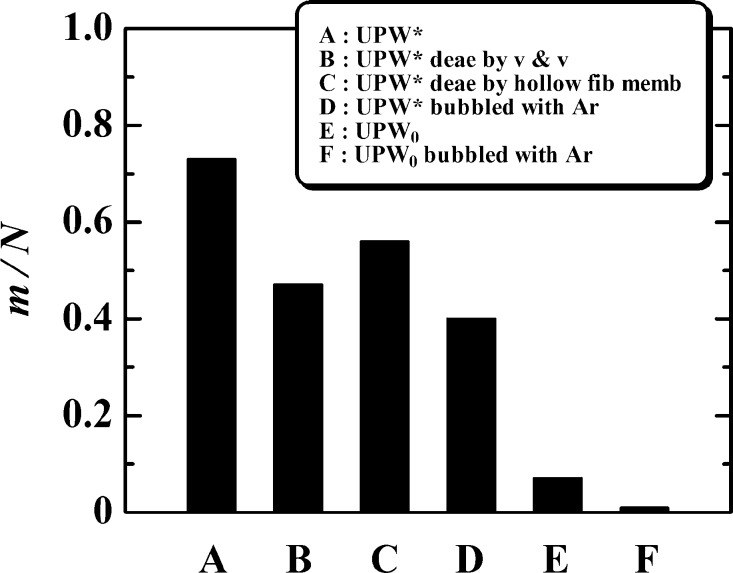
Effect of deaeration and Ar bubbling. A: *m*/*N* = 35/48 = 0.73, B: *m*/*N* = 9/19 = 0.47, C: *m*/*N* = 18/32 = 0.56, D: *m*/*N* = 8/20 = 0.4, E: *m*/*N* = 0.5/7 = 0.07, F: *m*/*N* = 0/12 = 0.

To confirm whether the membrane originated from the air dissolved in the water, we removed the dissolved air from UPW* by deaeration treatment or Ar-bubbling and conducted flow experiments. Deaeration was performed by vibration and vacuum (Figures 5(a)-1 and 5(a)-2) and by using a hollow fiber membrane and vacuum (Figure 5(b)). UPW* was bubbled with Ar to reduce the dissolved air (Figure 5(c)). The treated UPW* was stored in airtight conditions in a long tube (storage tube; Tygon 2001, plasticizer-free tubing, Saint-Gobain Performance Plastics Corp., USA; length: 3 m; inner diameter: 3 mm) to prevent air from re-dissolving in it, and the water flowed from the tube through the orifice (Figure 1(a)). Deaeration or Ar bubbling was quantified by measuring the dissolved oxygen (DO). DO in UPW* decreased from 8.2 to 1.4 mg/L by deaeration using vibration and vacuum at 25°C, from 9.1 to 3.0 mg/L by deaeration using a hollow fiber membrane and vacuum at 20°C, and from 8.5 to 2.1 mg/L by Ar bubbling at 23°C. Figure 6 shows that m/N was reduced from 0.73 for UPW* to 0.47 by deaeration using vacuum and vibration (UPW* deae by v & v), to 0.56 by deaeration using a hollow fiber membrane (UPW* deae by hollow fib memb), and to 0.4 by Ar bubbling (UPW* bubbled with Ar). However, the reduction in m/N from these treatments is not satisfactory. Therefore, we used another method to minimize the amount of dissolved air. Immediately after getting UPW_0_ from the UPW maker, we stored it in the storage tube that was directly connected to the outlet of the UPW maker. Then, we installed the storage tube in which UPW_0_ was contained between the vessel and the orifice (Figure 1). This procedure practically prevented the UPW_0_ from making contact with air before performing the experiments because the storage tube was long enough that the air could not penetrate from the reservoir to the UPW_0_ upstream of the orifice. We obtained the UPW_0_ for the flow experiment in this way. Furthermore, because the UPW_0_ was exposed to air for several seconds when installing the orifice in the experimental apparatus, the UPW_0_ was bubbled with Ar for 40 min to lessen the air resolution by the following process: evacuation in a flask (vacuum of = $-0.9\times 10^{5}$ Pa) (Figure 5(d)-1) → Ar filling of the flask (Figure 5(d)-2) → UPW_0_ injection into the flask (Figure 5(d)-3) → Ar bubbling of UPW_0_ for 40 min (volume flow rate of bubbling was 0.3 mL/s for 1000 mL of UPW_0_ in the flask) (Figure 5(d)-4) → transportation to long storage tube (Figure 5(d)-5), which differed from the process shown in Figure 5(c). This type of UPW_0_ is called UPW_0_ bubbled with Ar. m/N is 0.07 for UPW_0_ and 0 for UPW_0_ bubbled with Ar, as shown in Figure 6. These values are much smaller than 0.73 for UPW*. Figure 6 apparently shows that the membrane comes from the dissolved air. Airborne hydrocarbons absorbed on the orifice and reservoir surfaces [14], [15] may also be a candidate for membrane formation. However, we think this is not a main factor because the quantity of dissolved air strongly affected the membrane formation, as shown in Figure 6. This is mentioned again in Section 3-5, “*Irradiation with UV light*”.

### 3.4 Raman and IR spectra

**Figure 7:**
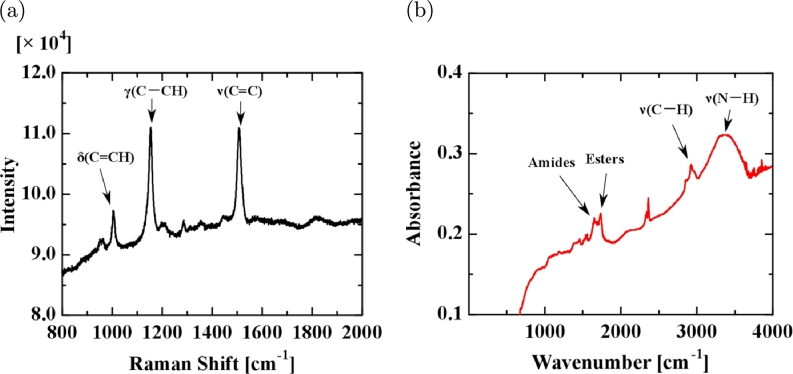
(a) Raman shift and (b) IR spectrum for the UPW* membrane.

Figures 7(a) and (b) show the Raman (XploRA, Horiba, Ltd., Japan) and IR (FTIR-8400S & AIM-8800S, Shimadzu Corp.) spectra for the UPW* membrane. Figure 7(a) of the Raman shift shows main sharp peaks at 1000, 1150, and $1510\;{\text{cm}}^{-1}$, characteristic of carotenoids, which have a linear, chain-like conjugated carbon backbone consisting of alternating carbon single (C—C) and double bonds (C=C) [16], [17]. Figure 7(b) of the IR spectrum shows peaks between 1500 and $4000\;{\text{cm}}^{-1}$ that may indicate organic compounds such as amides and esters (IR spectra for other UPW* membranes show the existence of sugars, amides, and esters; not shown here).

### 3.5 Irradiation with UV light

**Figure 8:**
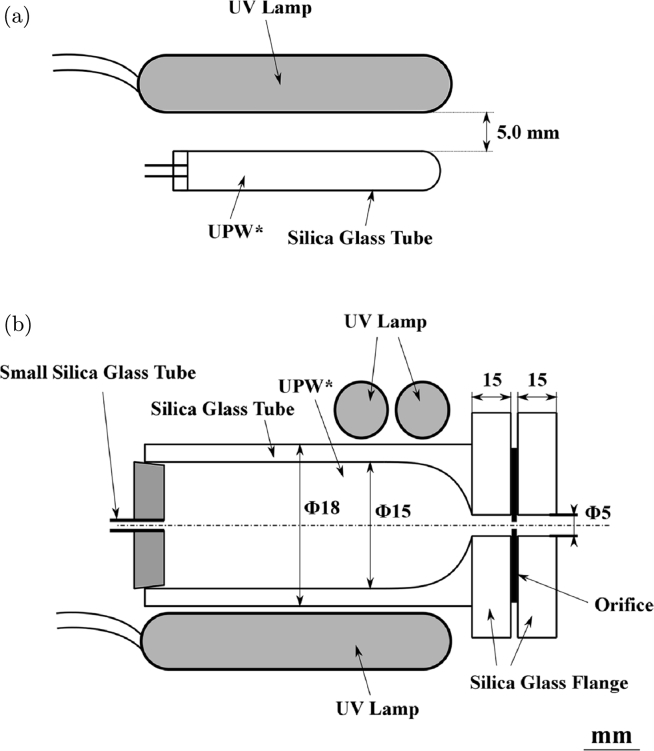
Methods of UV irradiation. (a) UPW* was stored in a silica glass tube (outer diameter: 33 mm, length: 200 mm) that passes through 185 and 254 nm UV light. The UV lamp had power of 40 W, diameter of 16 mm, and length of 200 mm, and it was a U-bubble lamp. The sample was irradiated for 3–4.5 h. The distance between the UV lamp and the silica glass tube was 5 mm. The UV-irradiated UPW* was transported to the piping system comprising the storage tube and silica glass tube in the orifice (Figure 4). (b) UV irradiation performed just before the flow experiment after setting the orifice in the apparatus. UPW* was stored in the silica glass tube (outer diameter: 18 mm, length: 110 mm) equipped with a silica glass flange that transmitted 185 and 254 nm UV rays. The silica glass tube was placed on the same UV lamp that was used in (a), and a second lamp was placed on the silica glass tube near the flange, perpendicular to the silica glass tube. After irradiation for 0.5–1 h, the flow experiment was started.

**Figure 9:**
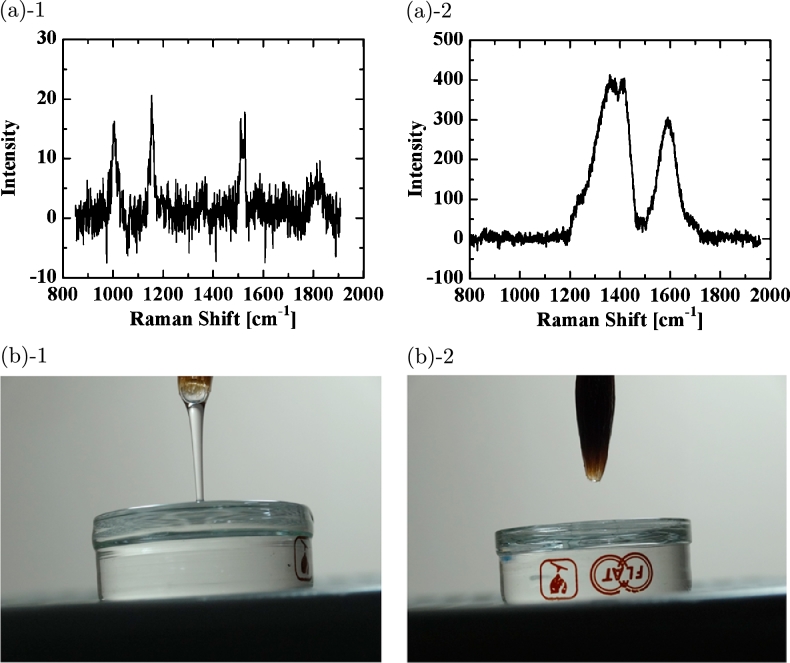
(a) Preliminary experiment using 0.01% AE(23) solution in UPW*. (a)-1 Raman shift of the membrane of the original non-UV-irradiated AE solution. (a)-2 Raman shift of the membrane of the AE solution UV-irradiated for 4 h. (b) Preliminary experiment using 0.01% PEO solution in UPW*. Photograph of PEO solution (b)-1 before and (b)-2 after UV irradiation.

For eliminating the organic compounds left in UPW*, UPW* was irradiated (Figures 8(a) and (b)) with UV light with wavelengths of 185 and 254 nm, which breaks most organic chains except C=O, C≡O, C≡C, N≡N, and C≡N [18], [19]. The UV lamp was placed near the tubes in methods (a) and (b) to ensure sufficient penetration of UV light into the water [20]. In method (a) (Figure 8(a)), UPW* filled in a silica glass tube (inner diameter: 30 mm) was irradiated using a UV lamp (Hybec Corp., Japan; 40 W, U-bulb type) for 3–4.5 h; then sent by Ar pressurization (Figure 5(a)-2) into the storage tube to prevent contact with air; and passed through an orifice attached to the test silica glass tube (inner diameter: 15 mm) (Figure 4). In method (b) (Figure 8(b)), UPW* was closed in the orifice-attached silica glass tube, where the orifice was attached to the end of the tube; irradiated with the same two UV lamps as in method (a) for 0.5–1 h just before beginning the flow experiment; and then passed through the orifice. We used method (b) to break the organic matter chains, irrespective of whether the organic matter was dissolved in water through water-air contact, left in UPW_0_, or came from the airborne hydrocarbons adsorbed on the orifice and reservoir surfaces. However, the difference in methods did not affect the experimental results. Conner-Kerr et al. reported that the irradiation time of UV (254 nm) on agar containing antibiotic-resistant bacteria of 120 s was sufficient to kill 100% of the bacteria [21]. In our case, water stored in glass tubes was irradiated with UV. Therefore, we confirmed the effect of UV irradiation by conducting two preliminary experiments using solutions of the non-ionic surfactant, polyoxyethylene (23) lauryl ether (AE(23); C_12_H_25_O(CH_2_⋅CH_2_O)_23_⋅H, MW = 1214.5), and polyethylene oxide (PEO; -(CH_2_CH_2_O)_*n*_-; MW: $4.5\times 10^{6}$) under similar conditions to the flow experiment. (a) Preliminary experiment used 0.01% AE(23) solution in UPW*. Figure 9(a)-1 shows the Raman shift of the membrane of the original non-UV-irradiated AE(23) solution, which contained a weak carotenoid pattern that was attributed to air dissolved in water. Figure 9(a)-2 shows the Raman shift of the membrane from the AE solution irradiated with UV for 4 h, which contained two peaks attributed to glassy carbon [22], [23]. The glassy carbon probably came from C generated by UV irradiation of the C—C chains. (b) Preliminary experiment used 0.01% PEO solution in UPW*. Figure 9(b)-1 shows PEO solution dripping from a brush before UV irradiation. Considerable spinnability was observed owing to the long C—C chain of PEO. Figure 9(b)-2 shows that the spinnability disappeared after UV irradiation, indicating that the C—C chains were broken. These results confirmed the effect of UV irradiation.

**Figure 10:**
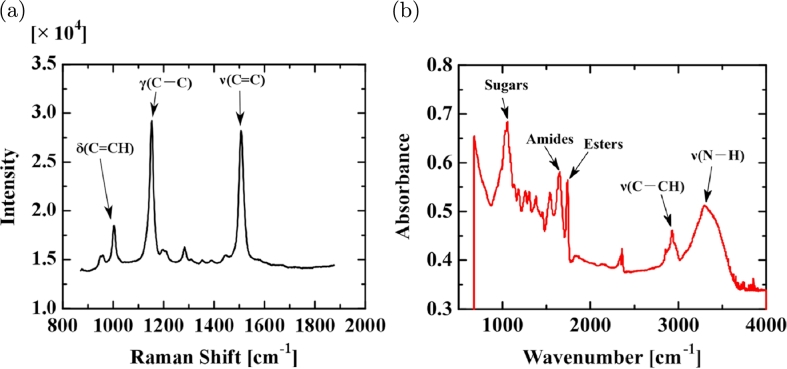
(a) Raman shift and (b) IR spectrum for the membrane from UV-irradiated UPW*.

Figures 10(a) and (b) show the Raman shift (NRS-3100, Jasco Inc., Japan) and IR spectrum (Nicolet iN10 Mx, Thermo Fisher Scientific Inc., USA) of the UV-irradiated UPW* membrane, respectively. Figure 10 (a) shows carotenoid peaks similar to those seen for UPW* (Figure 7(a)). Figure 10(b) shows an IR spectrum that contains several peaks that may arise from organic compounds similar to those in Figure 7(b) and sugars as well.

Consequently, the UV-irradiated UPW* generated almost the same membrane as non-UV-irradiated UPW*. This indicates the possibility that the organic matter of the membrane was synthesized from inorganic matter in the air dissolved in UPW*. For further investigation, we conducted a flow experiment using UPW_0_ bubbled with CO_2_.

### 3.6 UPW_0_ bubbled with CO_2_

**Figure 11:**
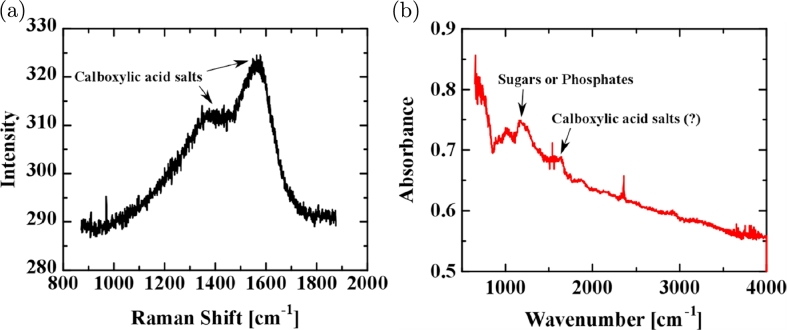
(a) Raman shift and (b) IR spectrum for the membrane from UPW_0_ bubbled with CO_2_.

We bubbled UPW_0_ with CO_2_ by the same method as that for UPW_0_ bubbled with air (Figure 5(d), pH of UPW_0_ bubbled with CO_2_: 4.1) and obtained a membrane, although the UPW_0_ bubbled Ar produced no membrane (Figure 6). Figures 11(a) and (b) show the Raman shift (NRS-3100, Jasco Inc., Japan) and IR spectrum (Nicolet iN10 Mx, Thermo Fisher Scientific Inc., USA) of CO_2_-bubbled UPW_0_, respectively. The spectra are smaller in magnitude than in Figures 7 and 10; however, they show the pattern of carboxylic acid salts. In other cases of UPW_0_ bubbled with CO_2_, the spectra showed a carotenoid pattern or mixed pattern of carotenoids and carboxylic acid salts (not shown). Figure 11 also suggests that the organic compounds in the membrane of UPW* are synthesized from the inorganic matter in the air in UPW*.

### 3.7 Inspection by PCM

**Figure 12:**
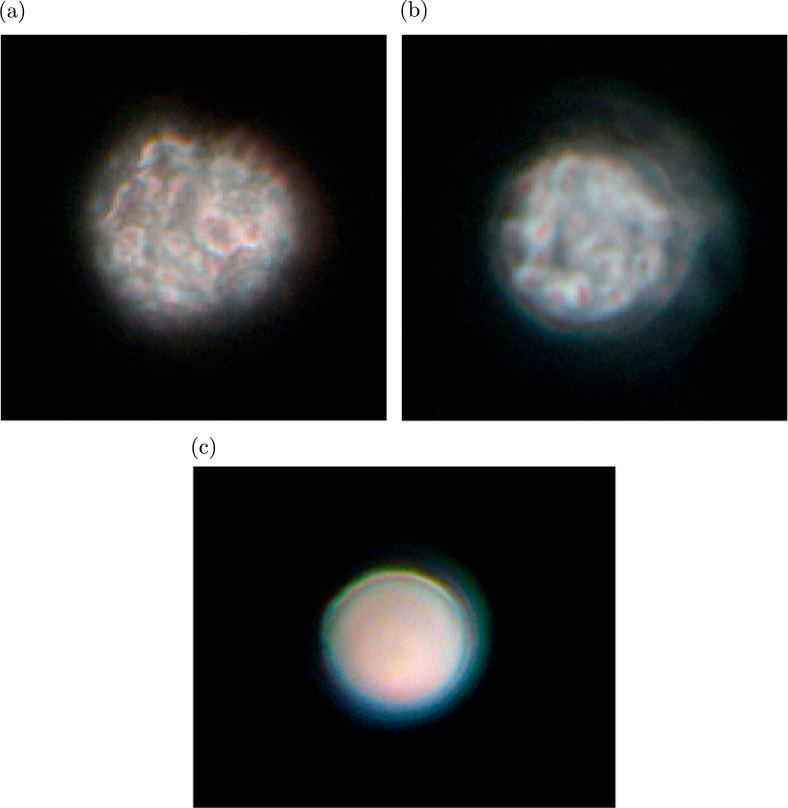
PCM images of (a) membrane from UPW*, (b) membrane from UPW* irradiated with UV, and (c) membrane from UPW_0_ bubbled with CO_2_.

We inspected the orifice after the flow stopped and examined the membranes found in the orifice by PCM. The PCM image in Figure 12(a), which corresponds to the spectra in Figures 7(a) and (b), is complicated and suggests that the components exist in a mosaic-like state in the membrane and that the red parts in the image are probably attributable to carotenoids. Figure 12(b) shows a PCM image of UV-irradiated UPW*, corresponding to the spectra in Figures 10(a) and (b), which is similar to Figure 12(a). Figure 12(c) shows a PCM image of UPW_0_ bubbled with CO_2_, corresponding to the spectra in Figures 11(a) and (b). Contrary to Figures 12(a) and (b), the image shows no complexity.

### 3.8 Effect of charges

**Figure 13:**
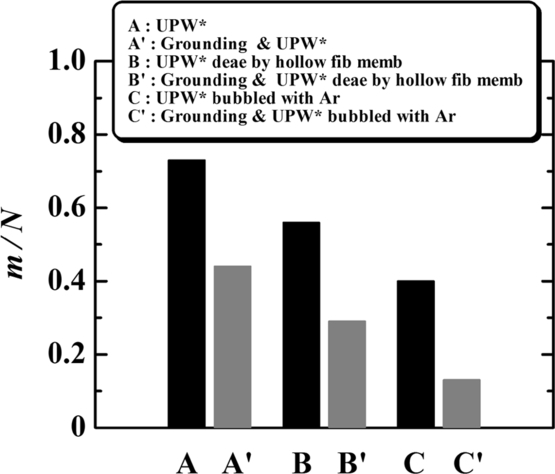
Flow properties of UPW*. Effect of electrically grounding the orifice. A: *m*/*N* = 35/48 = 0.73, A′: *m*/*N* = 8/18 = 0.44, B: *m*/*N* = 18/32 = 0.56, B′: *m*/*N* = 2/7 = 0.29, C: *m*/*N* = 8/20 = 0.4, C′: *m*/*N* = 1/8 = 0.13.

We examined the effect of the electrical grounding of the orifice on the probability of membrane formation. The three types of UPW* were passed through the grounded orifices. The result is shown in Figure 13 together with those for UPW* passed through nongrounded orifices: m/N without grounding (with grounding) is 0.73 (0.44) for UPW*, 0.56 (0.29) for UPW* deaerated by hollow fiber membrane, and 0.4 (0.13) for UPW* bubbled with Ar. Thus, m/N was decreased by grounding orifices for all UPW* used. This means that charges at the orifice wall are strongly related to membrane formation.

**Figure 14:**
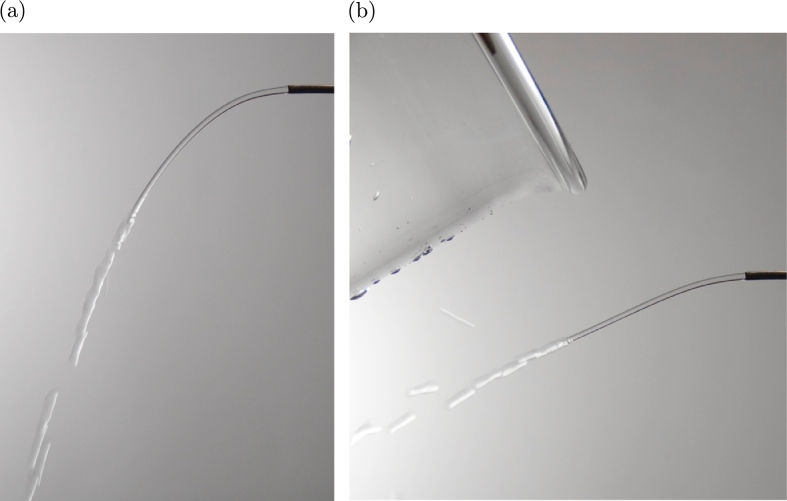
(a) Photograph of a jet of tap water flowing from a stainless tube with an inner diameter of 1.0 mm. The Reynolds number was 375, indicating that the flow was laminar. (b) Photograph of the jet attracted to positively charged glass, indicating that the water was carrying a negative charge.

Another experiment to demonstrate charges in water was performed using a jet of water ejected from a stainless steel tube (Figures 14(a) and (b)). The jet was attracted to positively charged glass, indicating that the water carried a negative charge and suggesting that the orifice surface was positively charged. In fact, positive voltage from water flow has been reported for an insulated pipe [24].

## 4 Discussion

**Figure 15:**
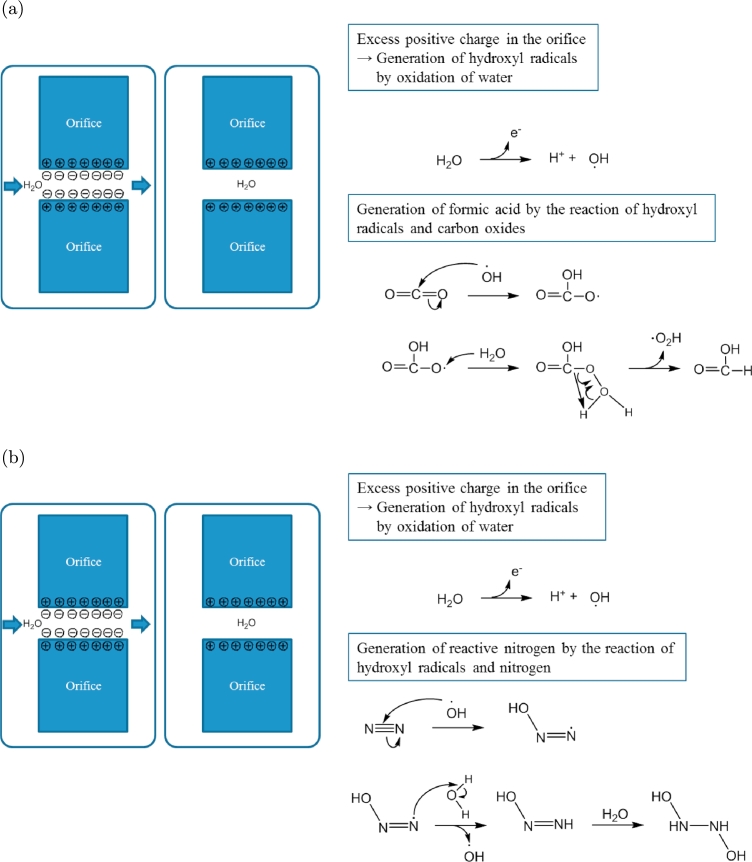
Process by which organic compounds are produced. (a) Formic acid and (b) Reactive nitrogen.

We consider that organic compounds are produced in the water after the flow through micro-orifices, as schematically shown in Figures 15(a) and (b). The water flow rubs the orifice wall, strips electrons from the wall, and carries them downstream of the orifice. The orifice, which is insulated, lacks electrons (negative charges) or contains excessive positive charges. Furthermore, metal ions dissolved in UPW from the orifice increase the excess of positive charges (Ni^+^ or Ti^+^ in the present study; Figures 3(a) and (b)). After the flow is stopped, the excess positive charges around the orifice wall strip water molecules of electrons (some of these electrons are used for making a metal membrane; Figure 3). This reaction converts the water molecules to hydroxyl radicals. The hydroxyl radicals continuously react with CO_2_ or N_2_ dissolved in water and produce two types of organic matter, formic acid and activated nitrogen, as schematically shown in Figures 15(a) and (b), respectively. Each of these goes on to build more complex organic materials, such as carotenoids, amides, esters, and sugars, for UPW* and UV-irradiated UPW*, as shown in Figures 7 and 10. Similarly, the reaction of hydroxyl radicals with CO_2_ may result in carboxylic acid salts, as shown in Figure 11.

Miller showed how organic molecules could have formed under primitive Earth conditions from inorganic precursors of methane, ammonia, water, and hydrogen by electrical discharge [25]. Submarine hydrothermal vents have been also been proposed as a possible origin of life [26]. The sonoanalysis of aqueous solutions containing acetic acid, methane, or carbon oxide in the presence of nitrogen gas produced several amino acids during ultrasonication [27]. Other sonochemical phenomena have been reported [28], [29]. Fallah-Araghi et al. reported a reaction-adsorption mechanism in micro-compartments that may be related to the current study [30]. In contrast to these studies, our results suggest that the conditions for organic synthesis are simply that water containing dissolved air flows through micro-orifices at ambient temperature. Thus, the result is related to the origins of life as well as to practical subjects.

## 5 Conclusion

In the present paper, we examined anomalies that accompanied the flow of water through micro-orifices. Two types of UPW were used: UPW that was exposed to air and in which air was dissolved (UPW*), and UPW that was kept from contact with air (UPW_0_). They were passed through Ni or Ti micro-orifices 20 μm in diameter at applied pressures of 50 and 1000 Pa. The flow velocities decreased and stopped over time. Membranes were frequently observed in the orifice. First, we examined the membranes by EPMA and found that chemical components of the membrane were as follows: C (∼60%), N (∼30%), O (∼4%), and Ni or Ti that was dissolved from the orifice metal. This composition was completely different from that of the original water. Next, to clarify the origin of the membrane, we examined three possible sources of the membrane material: material leached from the acrylic resin of the vessel; organic matter originally present in the water; and air dissolved in the water. The results showed that the membrane came from the dissolved air. Raman and IR spectroscopy revealed that the membrane comprised organic matter including carotenoids, sugars, amides, and esters.

After confirming the breaking of C—C chains by UV-irradiation in the preliminary experiments, UPW* was UV-irradiated. The chemical components of the membrane formed in the UV-irradiated UPW* and original UPW* were similar. Although UPW_0_ bubbled with Ar produced no membrane, UPW_0_ bubbled with CO_2_ produced a membrane that contained carboxylic acid salts.

Electric grounding of the orifice reduced the probability of membrane formation, and the jets issuing from an aperture bore negative charges. We assumed that the micro-orifices possessed positive charges generated by the flows.

We suggested that organic materials are synthesized in micro-orifices from the inorganic components in air or CO_2_ dissolved in water by the action of the hydroxyl radicals induced by flows.

## Declarations

### Author contribution statement

Tomiichi Hasegawa: Conceived and designed the experiments; Performed the experiments; Analyzed and interpreted the data; Wrote the paper.

Akiomi Ushida: Conceived and designed the experiments; Analyzed and interpreted the data; Contributed reagents, materials, analysis tools or data.

Masaki Goda, Yasushi Ono: Analyzed and interpreted the data.

### Funding statement

This work was supported by 10.13039/501100001691JSPS Kakenhi (Grant No. 23560189 and 26820041).

### Competing interest statement

The authors declare no conflict of interest.

### Additional information

No additional information is available for this paper.
